# Challenges and Opportunities in NUT Carcinoma Research

**DOI:** 10.3390/genes12020235

**Published:** 2021-02-05

**Authors:** Bin Gu, Maxwell C. Hakun

**Affiliations:** 1 Department of Obstetrics, Gynecology and Reproductive Biology, Michigan State University East Lansing, MI 48824, USA; 2 Department of Biomedical Engineering; Michigan State University East Lansing, MI 48824, USA; hakun-max@msu.edu; 3 Institute for Quantitative Health Science and Engineering, Michigan State University East Lansing, MI 48824, USA

**Keywords:** nut carcinoma, molecular oncology, cancer, cancer research

## Abstract

NUT carcinoma (NC) is a type of aggressive cancer driven by chromosome translocations. Fusion genes between a DNA-binding protein, such as bromodomain and extraterminal domain (BET) proteins, and the testis-specific protein NUTM1 generated by these translocations drive the formation of NC. NC can develop in very young children without significant accumulation of somatic mutations, presenting a relatively clean model to study the genetic etiology of oncogenesis. However, after 20 years of research, a few challenging questions still remain for understanding the mechanism and developing therapeutics for NC. In this short review, we first briefly summarize the current knowledge regarding the molecular mechanism and targeted therapy development of NC. We then raise three challenging questions: (1) What is the cell of origin of NC? (2) How does the germline analogous epigenetic reprogramming process driven by the BET-NUTM1 fusion proteins cause NC? and (3) How will BET-NUTM1 targeted therapies be developed? We propose that with the unprecedented technological advancements in genome editing, animal models, stem cell biology, organoids, and chemical biology, we have unique opportunities to address these challenges.

## 1. Introductions

**Figure 1:**
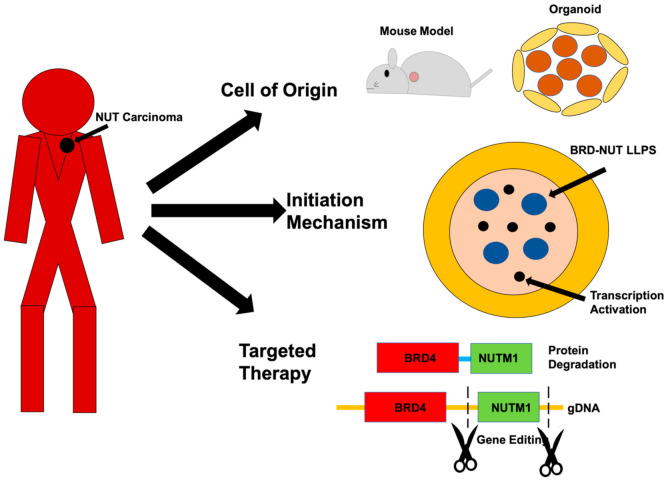
Challenges and opportunities in NUT carcinoma (NC) research. This review article raises three challenges in NC research: (1) What is the cell of origin of NC? (2) How do NUTM1 fusion genes cause NC? (3) How can we develop targeted therapies for NC? We propose these challenges can be effectively addressed now using new technological advancements in biomedical research, including genetically modified mouse models, organoids, imaging analysis of liquid–liquid phase separation (LLPS) and transcription, and targeted protein degradation and CRISPR-cas9 based gene therapy.

NUT carcinoma (NC) is an extremely aggressive cancer. In patients, it presents as a monomorphic low differentiated squamous cell carcinoma that arises primarily in any midline organs [1]. They are almost unequivocally resistant to currently available therapies, and the median survival time of diagnosed patients is about nine months, even with intense treatment. There is a clear need to understand the molecular etiology and potential means for targeted therapy for NC. In 1991, a cytological defect with a t(15;19) chromosome translocation was first described in two separate cases [2]. It was then reported in 2003 that the t(15;19) translocation generated a fusion gene between bromodomain and extraterminal domain (BET) protein 4 (BRD4) and a testis-specific gene NUT midline carcinoma family member 1 (NUTM1) [3]. In the following years, other fusion genes all involving the NUTM1 gene, including BRD3-NUTM1 [4], NSD3-NUTM1 [5], CIC-NUTM [6], and ZNF532-NUTM1 [7], have been associated with the development of NC. It is known that the NUTM1 fusion genes can be associated with NC development in even very young children without significant accumulation of other somatic mutations, suggesting a strong causal effect of these genes in NC development [8]. This strong causal effect provides a unique opportunity to unravel the molecular mechanism of NC development and presented the NUTM1 fusion protein as an ideal target for therapy. Indeed, research on tumor samples and primary cells from patients, as well as ectopic expression systems in cell lines, have provided tremendous knowledge on the molecular changes that were induced by the NUTM1 fusion proteins and points to promising avenues for targeted therapies, including induced differentiation therapy and BET targeted therapies [9]. However, in spite of this research, a few fundamental questions still remain unanswered as to the etiology of NC and the potential methods for targeted therapy. We try here to raise three challenging questions and suggest new ways to address them with new technological advancements in biomedical research (Figure 1). Since the majority (70%) cases of NCs are associated with the BRD4-NUTM1 fusion gene, we will refer to the NC causing fusion genes as BRD4-NUTM1 unless specifically describing other fusion genes such as ZNF532-BRD4.

## 2. What Is the Cell of Origin of NC?

**Figure 2:**
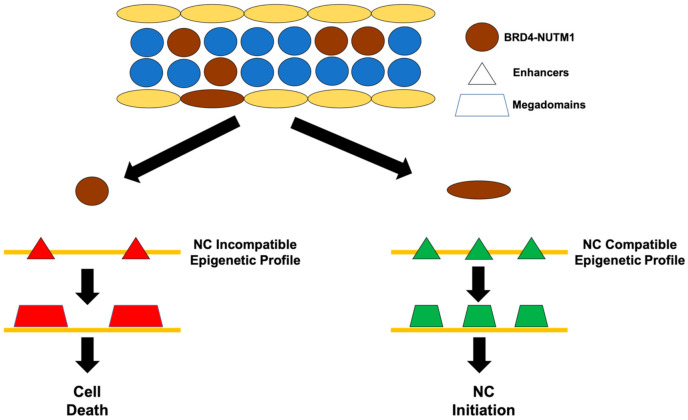
A model of the cell of origin of NC. Most cell types (**left**) possess an epigenetic profile that is not compatible with bromodomain and extraterminal domain BRD4-NUTM1 expression. When the BRD4-NUTM1 fusion proteins are expressed in these cells, it drives the establishment of acetylated histone megadomains that are not compatible with cell survival and causes cell death. Some unknown cell types (**right**) possess an epigenetic profile that is compatible with BRD4-NUTM1 expression. When the BRD4-NUTM1 fusion proteins are expressed in these cells, it drives the establishment of acetylated histone megadomains that cause cell transformation and NC formation.

Several lines of evidence suggest that NC might originate from a rare cell population preferentially in midline epithelial tissues. These cells can be with some undifferentiated features or can be reprogramed to an undifferentiated status (Figure 2). There are three observations that suggest this line of thinking: (1) Unlike many solid tumors that are likely polyclonal and resulted from the accumulation of mutations throughout a long period of time of the patient’s life [10,11,12], NC seems to be largely driven by the single BRD4-NUTM1 forming translocation with minimum mutation loads in oncogenes and tumor suppressor genes [13]. Also, NCs generally affect younger patients (with a median age of occurrence at 24 years old) and can even occur in infants [14,15]. These suggest that the oncogenic process of NC is likely more resemble pediatric leukemia, which is typically monoclonal rather than the polyclonal nature of common solid tumors [1]. (2) NUTM1 fusion proteins are cytotoxic when ectopically expressed in cell lines but can induce NC in patients [9,16]. This may be related to the tissue-specific establishment of histone acetylation mega-domains where only the correct combination of pre-existing enhancers permits cell survival and transformation under NUTM1 fusion gene expression to NC [17]. Combined with its rare prevalence, it appears that NC arises in small populations of epithelial cells located in the midline organs, probably with some undifferentiated qualities [18]. (3) NCs are low differentiated squamous carcinomas suggesting they possess undifferentiated properties [18]. NC typically appears and is classified as a poorly differentiated carcinoma. In histological studies, NCs are defined as squamous carcinomas with poor characterization. A distinguishing feature of NC is their monomorphic nature consisting of sheets of similar cells, unlike the pleomorphism seen in other carcinomas. Critically, when treated with drugs such as histone deacetylase (HDAC) inhibitor or BET inhibitors, NC cell lines can be induced to express markers of terminally differentiated squamous epithelial cells [9]. Furthermore, it has been shown that a stem cell marker Sox2 is expressed in NC cell lines and required for their proliferation [19]. These observations imply that NC cells are trapped in an undifferentiated status, either from the cell type that they originated from or from a reprogramming process that BRD4-NUTM1 has induced in a differentiated cell type. As has recently been illustrated in brain cancers, a clear understanding of the developmental trajectory of cancer development can provide not only a fundamental knowledge of the mechanisms of cancer formation but also expose specific vulnerabilities of the cancer cells for therapy development [20,21,22].

The identification of the cell of origin of NC has been challenging due to several limitations: (1) most patients are diagnosed at a relatively late-stage of NC development; thus, information that indicates the early origin cells may not be easy to retrieve; (2) NUTM1 fusion proteins drive radical reprogramming of the epigenetic status and transcription profile of the expressing cells [17]; therefore the developmental trajectory and origin cell type may not be easily inferred by the common methods of clustering transcriptional profiles, and (3) because ectopic expression of NUTM1 fusion proteins cause cell death in most in vitro systems, even inducible expression systems may not be informative for inferring the original cellular status for NC initiation. The key to defining the cell of origin of NC is, therefore, a model system that can recapitulate the earliest events of NC initiation and isolate the originating cells for analysis. Conceptually, the NC causing chromosome translocation will be induced at the clonal frequency in a heterogeneous cell population recapitulating the tissue context of NC formation. By natural selection, most cells in which the translocation occurs will die, and only the cell with a conduit status for NC formation will survive and expand. These cells will carry some kind of reporters that allow their isolation and analysis. We believe the technological advancements over the last decades has provided all the tools necessary to establish such a model. First, a chromosome engineering technology that utilizes cre-loxp induced chromosome translocation in embryonic stem cells and knock-in mouse models was developed about 20 years ago and has been successfully used to model the initiation process of fusion gene-mediated cancers such as myeloid leukemia [23,24,25]. The recent advancement of CRISPR-Cas9-mediated genome editing technology has made engineering the cell or mouse line required for the inducible chromosome translocation much more efficient and rapid [26]. Second, over the last few decades, numerous genetically coded reporter systems for fluorescent, bioluminescent, and positron emission tomography (PET) imaging that can be used to trace cancer progression and fusion gene expressing cells have been developed and provide the capability to sensitively detect and isolate NC originating cells [27,28,29]. The recent advancement of CRISPR-Cas9-mediated large fragment knock-in technologies, including the 2C-H-CRISPR technology developed by our group, has made engineering the reporter models more efficient and rapid [30,31]. Furthermore, at least two sophisticated systems could be used for modeling the initiating cell population and tissue context for NC. The first is using genetically modified translocator mice, which has already proved valuable to identify the developmental origin of leukemias [32]. Although there are drawbacks stemming from potentially different gene regulatory networks between mice and humans, mice have been proven to be conserved enough to humans to provide important understanding for human diseases over several decades. In fact, until recently, animals were the only models to recapitulate complex cellular interactions in tissue and still remain largely the only way to recapitulate all aspects of the environment of cancer formation, including the vascular, neurological, and immunological environment. They are arguably still one of the most promising ways to model NC initiation and identify the originating cells. Second, organoid technology has recently emerged as a promising way to model processes involving the interaction of multiple cell types in complex human tissue [33]. The main advantage is that an organoid from human stem cells can, to a certain extent, recapitulate the cell type heterogeneity and architecture of a human organ and preserve the human gene regulatory networks. Indeed, human intestine organoids have recently been successfully applied to model the distinct oncogenic mechanisms and developmental trajectory in cancers caused by a number of oncogenic mutations, as well as to test experimental therapies [34,35,36]. Critically, organoids modeling organs relevant to NC, including the esophagus and lungs, have been recently reported and could thus be further developed to model the initial steps of NC formation and identify NC originating cells [37,38]. However, it remains a question whether these organoid systems could recapitulate all cell types in a tissue, including the rare stem cell from which NC arises. Thus, a complementary approach combining the unique strengths of the mouse model and organoid systems may be a more optimal way of success. With these promising new technologies, we expect significant new development in mouse and organoids models in NC research and a clear definition of the NC cell of origin in the not-too-distant future.

## 3. How Do NUTM1 Fusion Genes Cause NC?

A prominent theory on oncogenesis suggests that oncogenic mutant gene products radically change the gene regulatory program of normal cells to initiate cancer. In many cases, the radical change comes from the accumulation of numerous oncogenic mutations, each contributing small effects over many years of life. However, NUTM1 fusion genes can nearly act as the sole factor in driving oncogenic transformation, indicating an extremely strong reprogramming capability on cellular gene regulation. This reprogramming capability may be attributed to the native function of both fusion partners. The role of the BET portion of the fusion genes seems to be clearer and more related to their chromatin binding properties. BET proteins are acetyl-histone binders that can bind broadly to accessible regions of chromatin such as enhancer regions [39,40,41]. This property likely provides a broad chromatin targeting mechanism that is critical for the BRD4-NUTM1 fusion proteins to reprogram the cellular genome. Indeed, even when BET proteins are not part of the fusion gene, for example, in the case of ZNF532-NUTM1, the fusion protein still targeted the chromatin by a molecular interaction between ZNF532 and BRD4, further supporting the critical chromatin targeting roles of BET proteins in NC formation [7]. The reprogramming activity of NUTM1 fusion proteins, on the other hand, is more enigmatic and likely lay in a not well-characterized function of NUTM1 during spermatogenesis.

**Figure 3:**
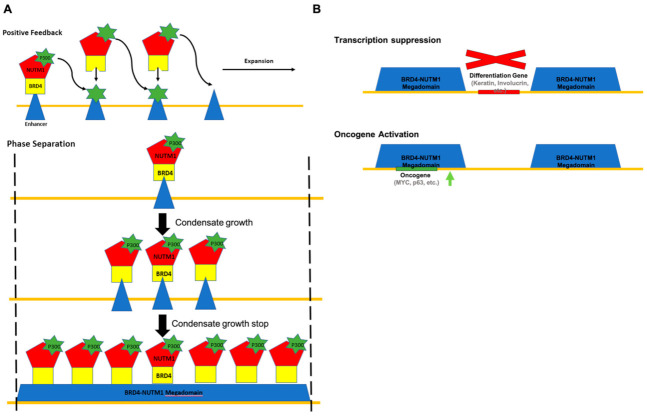
Models of NC initiation mechanisms. (**A**) Two possible mechanisms for the establishment of acetylated histone megadomains. Top: the formation of megadomains through P300-mediated positive feedback. The boundaries of megadomains are defined by currently unknown biochemical barriers. Bottom: the formation of megadomains through NUTM1-mediated phase separation and condensate growth. The boundaries of megadomains are defined by condensate size that is determined by the nuclear concentration of BET protein 4 (BRD4)-NUTM1. (**B**) Two possible mechanisms for megadomain-mediated oncogenesis. Top: megadomains drive NC formation by suppressing the expression of differentiation promoting genes. Bottom, megadomains drive NC formation by suppressing the expression of oncogenes.

Immunofluorescence analysis in NC tumor tissue, primary NC cells, and cell lines ectopically expressing the BRD4-NUTM1 fusion gene demonstrated unequivocally that the NUTM1 fusion proteins form large foci in the cell’s nuclear region [42]. These foci always colocalized with large clusters of acetylated histone signals. More recently, it has been shown that NUTM1 fusion proteins can induce very large, acetylated histone domains that sometimes fill the whole topological associated domain (TAD) [17]. Knock-down and chemical BET dissociation experiments demonstrated that the existence of these large, acetylated histone domains depends on the expression of the BRD4-NUTM1 fusion proteins and are required for the proliferation and undifferentiated status of NC cells. Importantly, when NUTM1 fusion genes were overexpressed in cell lines, these large, acetylated histone domains seemed to prominently form around the existing enhancers of the host cell, again corroborating the chromatin targeting role of the BET portion of the fusion gene. Despite this, how the histone acetylation expands from a well-defined enhancer region to the “mega” domains is a challenging question to answer. The hint comes from NUTM1’s native function in spermatogenesis. In order to package the whole-genome into the sperm head, which is approximately 1/7th the size of any somatic cell nucleus, almost all the histones on chromatin are replaced by the small and highly charged protamine protein during spermatogenesis [43]. Before this replacement, histone H4 in spermatocytes is globally modified by acetylation. This broad histone acetylation process is mediated by the testes specific protein named NUTM1 [44]. Presumably, through its self-multimerization (and possibly phase separation) capability due to its highly disordered protein structure, NUTM1 forms large clusters on the chromosome of spermatocyte that recruit p300 acetyltransferase and establish broad histone acetylation domains. These domains will then be removed and replaced by protamine. It has not been defined how NUTM1 binds to the sperm chromatin in the first place; however, presumably, some unknown proteins help NUTM1 to target the chromatin. In the situation of NC, it was proposed a similar process led to the establishment of the “mega” acetylated histone domain [17]. Essentially, the BET portion of the NUTM1 fusion protein fulfills the chromatin targeting function and brings the fusion proteins to existing acetylated histones on the chromatin. NUTM1 then recruits the histone acetyltransferase (HAT) P300 to acetylate nearby histones, then that acetylated histone will recruit more NUTM1 fusion proteins and let the process propagate as a positive feedback process. This model nicely explains the mechanism of the expansion of the acetylated histone domain but faces challenges explaining how this feedforward loop stops. Because it was observed that the “mega” acetylated histone domains do have boundaries and do not normally fill the whole chromosome, there must be an explanation of why the feedforward loop stops at these boundaries. Since the boundaries often overlap with the boundary of TADs, one explanation could lay in certain strong insulating properties originating from the biochemical complex at domain boundaries such as CTCF complexes; however, this requires further investigation [17]. An alternative explanation may lay in the unique biochemical nature of the NUTM1 protein (Figure 3A). Recently, it has become more accepted that proteins with intrinsically disordered sequences have the ability to go through liquid–liquid phase separation (LLPS) and form membrane-less condensates on the microscopic scale [45]. Intriguingly, NUTM1 protein is mostly composed of intrinsically disordered sequences. Therefore, it can be envisioned that the microscopically visible foci formed by NUTM1 fusion proteins in NC cells are a type of these membrane-less condensates. In this scenario, the NUTM1 fusion proteins occupancy domains, and thus the acetylated histone megadomain, expands by LLPS seeded by the first DNA-binding of the fusion proteins on endogenous enhancers rather than the positive feedback loop previously proposed. Because the size of LLPS condensates is limited by the available concentration of their components, in this case, the NUTM1 fusion proteins and the biochemical environment of the cell nucleus, this mechanism can set a limit on the size of the fusion protein occupancy domain and the mega-histone acetylation domain, which may be more consistent with the experimental observations. Critically, technologies are now available to test these models. Live-cell fluorescent imaging and optogenetic technologies have advanced significantly to analyze the LLPS behaviors of intrinsically disordered proteins [46,47]. These advancements have already led to new fundamental understandings of the mechanisms of RNA processing, chromatin structure organization, transcription control, and endless other biological processes. These new understandings have also offered a new mechanistic understanding of human diseases [48]. All these technologies can be used to analyze the forming process of the NUTM1 fusion proteins’ occupancy domain and thus the acetylated histone megadomain. Moreover, since the nature of the LLPS process is extremely sensitive to the concentration of condensate components, it is important that cells expressing the NUTM1 fusion proteins and reporters have similar expression controls as in NC cells. Here again, the advancements in genome editing technologies for engineering models with physiological expression levels of NUTM1 fusion proteins will provide strong support for researchers.

Another challenging and, in fact, controversial problem is how the downstream mechanism induced by the global epigenetic reprogramming described above is responsible for the oncogenic activity of NUTM1 fusion proteins. Two not mutually exclusive hypotheses were proposed: (1) The transcription suppression hypothesis suggests that the histone acetylation megadomain sequesters transcription factors and epigenetic factors such as HAT away from genes critical for cellular differentiation [42] (Figure 3B). Because of that, the NC cells fail to activate squamous differentiation genes and tumor suppressor genes and are trapped in an undifferentiated proliferative status. Supporting this hypothesis, it has been reported that treating NC cell lines with HDAC inhibitors can recover the expression of terminal squamous cell differentiation genes and induce differentiation and cease proliferation [49], presumably by reestablishing the histone acetylation and expression of these genes. (2) the oncogene activation hypothesis suggests that the histone acetylation megadomain can activate oncogenes such as Myc when formed over them. This is supported by findings that the BRD4-NUTM1 occupancy domain and histone acetylation megadomain in NC cell lines overlaps with oncogenic loci, including the Myc loci and correlate with the expression of oncogenesis associated genes such as *P63*, *MYB* and *MED24* [17]. Furthermore, it has been reported that Myc is downstream of BRD-NUTM1 fusion proteins and whose control is necessary and sufficient for the blockage of differentiation in NCs [50]. A very recent report on the activity of P300 HAT inhibitor for NC treatment and the suppression of the expression of oncogenes such as Myc and P63 also supports this second hypothesis [51]. Both of these hypotheses imply that the oncogenic function of NUTM1 fusion proteins is highly dependent on the position of the histone acetylation megadomain they induce, and therefore consistent with their cell type restricted oncogenic potential. However, they point to different routes for developing therapies, one utilizing induced differentiation by transcriptional activation, another using oncogene inhibition by transcription suppression. Noticeably, both of these hypotheses are formulated based on observation in a bulk NC cell population and could both be true in distinctive subpopulations of cells. Due to the heterogeneity of cancer cells, before committing to any one of these hypotheses, one needs to be certain about which and how much each mechanism holds true in a single-cell in NC. The experiments to test these hypotheses at a single-cell level can now be conceived with a few recent technological analyses. The first critical technological advancement is single-cell multi-omics [52]. It is now possible to analyze the transcriptome of a single-cell at the same time with some of its epigenetic properties by genomic sequencing. Although right now it is still not possible to define the distribution of a specific epigenetic mark such as acetyl-histone and the transcriptome in the same single-cell, we could expect feasibility soon with the rate of development in single-cell genomics technology. With these developments, we will be able to finally correlate the global epigenetic and transcription changes induced by the NUTM1 fusion proteins at the single-cell level. However, before that, live-cell imaging technology can still offer critical insights. It is currently possible to visualize the whole information flow of transcription by live imaging. The binding of general transcription factors and RNA pol II complexes can be measured by single molecular tracking technology by which a single copied locus in a genome can be localized by different versions of advanced CRISPR-Cas9-dependent imaging technology and the production of nascent transcripts–transcription bursts can be measured using hairpin-based imaging in single-cells [53,54,55]. A combination of these technologies can be used to test these hypotheses in single-cells of NC cell populations and reveal fundamental new knowledge.

## 4. How Can We Develop BET-NUTM1 Targeted Therapies?

Up to date, the clinical treatment for NC has not been very successful. The state-of-the-art treatments of NC currently include surgical resection with or without adjuvant radio or chemotherapy [56,57]. However, cancer often responds initially to treatments but then develop therapy-resistant relapse. The median survival time of diagnosed patients is about six months, even with intense treatment. Since NCs are likely driven solely by the BRD4-NUTM1 and other NUTM1 fusion proteins, targeted therapy against them are promising routes for therapy development. In addition to serving as a single therapeutic agent, given the recent new understandings on the immune micro-environment of NC [58], these targeted therapies can also be combined with chemotherapies and immune-therapies such as PD-L1 targeted therapy to achieve more beneficial outcomes in the future [59,60].

Since NC can be viewed as caused by a global epigenetic reprogramming involving histone acetylation largely driven by a single NUTM1 fusion protein, there are several promising routes for targeted therapy. The first targeted therapy that has been tested targets histone acetylation based on the hypothesis that the mega-acetylated domains generated by NUTM1 fusion protein expression trap HATs in these domains and result in global histone hypoacetylation in most parts of the genome. This causes the suppression of squamous differentiation genes in NC cells and traps them in an undifferentiated status with uncontrolled proliferation. With this logic, histone deacetylase (HDAC) inhibitors have been tested as targeted therapy agents. HDAC inhibitors presented encouraging effects in inducing the differentiation of NC cell lines and preventing rapid proliferation, suggesting it can partly reverse the NC phenotype [49]. Despite this, small scale clinical trials with an FDA approved HDAC inhibitor showed only limited benefits in patient survival [49,61]. As discussed in the previous section, it is not clear whether differentiation arrest is the only reason for NC phenotype in vivo; therefore, HDAC inhibitor may only target a portion of the NC defects. Furthermore, as an agent targeting global histone acetylation, HDAC inhibitors are expected to show more deleterious side effects [62]. Indeed, a patient in the clinical trial discussed above had to terminate the treatment due to significant side effects in the form of moderate gastrointestinal toxicity, fatigue, and reversible grade 3 thrombocytopenia. These adverse side effects could complicate the outcome of using HDAC inhibitors as a targeted therapy for NC [49]. Given the great success in drugs targeting cancer-driven fusion proteins such as Gleevec (targeting BCR-ABL in chronic myeloid leukemia) [63,64], directly targeting the NUTM1 fusion proteins themselves is another promising avenue for targeted therapy. Critically, BET proteins such as BRD4, which is part of a large portion of NUTM1 fusion proteins (>70%), have been widely studied as a therapeutic cancer target [65]. There are many BET inhibitors (iBETs) available or under development [65]. This led to multiple preclinical studies and clinical trials using iBET to treat NC with promising outcomes, including differentiation and ceased proliferation of NC cell lines and a partial response in patients. In a study published in 2010, treating NC cells with the iBET JQ1 was shown to cause the dissolution of BRD4-NUT nuclear speckles. JQ1 also induced rapid terminal differentiation, apoptosis, and arrested growth of NC cells in vitro, and induced tumor growth suppression and improved survivability in an NC xenograft model [66]. Birabresib (OTX015) is an iBET currently being tested clinically [67]. A study of four late-stage patients treated with birabresib marked a quick response to treatment, with two patients reporting symptomatic improvement and another stabilization of their disease. Three of the patients exhibited survival times of 17, 18, and 19 months with the only reported side effects being moderate gastrointestinal toxicity and fatigue and reversible grade 3 thrombocytopenia. However, they all developed treatment-resistant cancer after several months after iBET treatment through a hitherto unknown mechanism [67]. Two critical problems here still need to be addressed. First, most of the iBETs function by partially disengaging the bromodomains in BET proteins, which decreases their DNA binding. It is important to mention, however, that it has been shown BET proteins may play additional roles in cancer than its DNA binding activity [68]. Indeed, recently BET proteolysis-targeting chimera (PROTAC) called dBETs have been developed that can degrade the BET proteins in a targeted way [68,69]. These dBETs have shown much stronger cytotoxic activity against cancer cells in vitro and in xenograft assays. Thus, dBETs can be promising agents for NC targeted therapy. However, the potential pleiotropic effect of any BET targeting therapy agents could pose the second significant challenge. Given most of the BET targeting agents cannot discriminate different BET proteins, including BRD2 and BRD3, they also certainly cannot discriminate the normal BET proteins that play important physiological roles from the BET-NUTM1 fusion protein. Therefore, one can easily imagine detrimental side effects of BET targeting drugs in patients, even more so for dBETs than iBETs. Intriguingly, the NUTM1 portion of the fusion protein may actually be a very promising target for therapy due to its function and tissue distribution. NUTM1 is only expressed in testes under normal conditions; therefore, any targeted agents against NUTM1 are expected to only target the fusion protein in somatic cells and thus present minimum physiological side effects. Since NUTM1’s only primary function seems to be in ensuring normal spermatogenesis, the only expected side effects may be a fertility defect in male patients after treatment [44]. However, with highly established technologies in sperm storage and assisted reproduction, these fertility defects can be relatively easily addressed. Moreover, because of the existence of the blood-testes barrier, one can carefully design the chemical or delivery agents to avoid distributing the targeted agents to the testes, therefore protecting spermatocytes. With these considerations, agents with very strong effects against NUTM1 may be used to target NC, such as PROTACS and even NUTM1 gene disrupting gene therapy. With the recent developments in both PROTAC technology and in vivo CRISPR-Cas9-mediated gene therapy technology [70,71], these promising avenues can soon be pursued. In order to develop these promising targeted therapies, appropriate preclinical models that recapitulate at least certain levels of the tissue and possess systemic complexity would be needed. The genetically modified mouse models and organoid models discussed in Section 1 could prove critical for these endeavors. Furthermore, because the protein structure of NUTM1 is currently unclear, it will likely require significant efforts to design and develop targeting agents such as nanobodies or small molecular ligands to direct the specificity of PROTACs [70]. Similar levels of effort and investment will also likely be required to develop CRISPR therapies. Therefore, before committing to these efforts, a proof of principle study will likely be needed to demonstrate the efficacy of removing the BET-NUTM1 to treat NC in a model close to in vivo conditions. Recently, developed genetically encoded degron systems such as dTAG and the Auxin-inducible degradation system, which offer controlled degradation of endogenous proteins, will prove highly valuable for this purpose [72,73,74]. Combining these degron technologies and CRISPR-Cas9-mediated large fragment knock-in technologies, mouse and organoid models can be generated to test targeted BET-NUTM1 degradation. With all these technological advancements, we believe that now is the start of an exciting time for the development of targeted therapy for NCs.

## 5. Conclusions

NCs are devastating cancer. By integrating technology advancements in many frontiers, the critical challenges discussed above can be effectively addressed. A bright future will soon be seen for understanding NCs and treating NC patients.

## Data Availability

Not applicable.
